# Modern Sociology

**Published:** 1902-02-15

**Authors:** 


### Modern Sociology.

### SPECIAL SCHOOLS UNDER THE LONDON SCHOOL BOARD.

### I.—Introductory.

The other day a lady visited a patient in a lunatic asylum, and although she knew the weather was dull and foggy, she could not for days afterwards dismiss the notion that it was a brilliant sunshiny morning; the effect of cheerful wall-paper, flowers, singing birds, and pretty things at that asylum blotted out everything else. We have much the same feeling about our visits to the special schools under the London School Board (with one exception, to which we shall refer presently); and we should like to prescribe some such visits, by way of mental tonic, for those who are inclined, as Stevenson says, to bleat about their own sorrows and the burthen of life.

The children dealt with in these classes are (1) blind, (2) deaf, (3) mentally deficient, and (4) cripples. They are grouped in mixed classes, boys and girls, according to capacity; the Board lays down guiding lines as to curricula, but naturally much more elasticity is necessary than in the case of normal classes; and we have found that among head teachers, at any rate, there is plenty of room for invention, and great scope for individual resource. One lady writes simple songs for her children tô sing, and has taught them more of botany than many normal children know, through half holidays in a South London Park, acorns grown in the schoolroom window, and leaves and berries collected by the children.

The drawings by the deaf children show promise of real talent for designing; and here again there is great scope for the teachers. It is very pleasant to observe the spirit of enthusiasm which actuates them; to notice their untiring efforts to develop the very limited mental or physical capacities of the children; their varied experiments, and their pleasure at the results—results sometimes which would seem a hopelessly inadequate reward to the ordinary teacher of normal pupils. It may safely be said that only those teachers capable of feeling this enthusiasm are the least use here; and it is evident that the greatest possible care is exercised in their selection. Inexhaustible patience is required, a power of sympathy; in a word, a love for these children who are so heavily handicapped in the race, else the work is bound to be a failure; and there seems to be a hopeful opening in these schools for young women of the upper classes (we use the phrase for want of a better) with a taste for individual teaching, and a desire to put into practice that spirit of "zeal for man" which we are told is in the air. That the unconscious influence of the teacher is a very real factor in education would, one would imagine, be sufficiently obvious. We have a vivid recollection of a certain elementary school in a country village, where all the teachers, from the master downwards, had something almost repulsive about their personal appearance—either front teeth at acute angles, or goggle-eyes with enormous glasses. One cannot, of course, eliminate a man or woman otherwise fitted for educational work merely on account of an accidental resemblance to the Mad Hatter, and we suppose it would be beyond the scope of the Board of Education to provide free dentistry at the training colleges; but something might be done in the way of pointing out to teachers the simple truth that personal appearance plays no small part in their influence upon their pupils. We have learnt to decorate the schoolroom walls with something more artistic than the mere map; in the special schools, at any rate, there are always flowers, and in some canaries and other singing birds in cages, or an aquarium with gold fish; and a pretty teacher, tastefully clothed (we saw one with an art student's pinafore over her dress) must greatly add to the pleasant impression a child has of its schoolroom. And if this is so with the abnormal child, is it not equally true of the one whose faculties are even more on the alert, and who is photographing impressions on its brain moment by moment?

There can, perhaps, be no greater or sadder contrast than to go from such a school as we have indicated, where all is bright and cheery, and the children are eager to answer the questions of the teacher—the little lip-readers crowd round, each doing his best; the mentally deficient in their baby-lessons vie with each other in naming a letter or folding a paper square, and the cripples, some more than normally bright, show evident signs of enjoyment. In the schools, as at present staffed, the blind are leading the blind in a very literal sense. In the one we have just visited, there are three classes, and no sighted teacher. Some of the children can see a little; one teacher can distinguish colours. The lifelessness, apathy, and stillness of these three classes lingers as an unpleasant memory. We were shown their knitting and baskets—very creditable work indeed—by teachers who had to grope their way to the cupboards where they were kept, and who turned sightless eyes towards a corner where a little rustling or whispering told of inattention. Meanwhile, the children's hands were dirty; one baby, with terrible eyes, was apparently asleep on its desk, its head tied up on account of earache. In the eldest class we watched a boy and a girl type-write; both touched the right keys in most words, but the writing was squeezed into a corner of the paper, and the teacher could not possibly correct the mistakes in spelling and alignment, once they were made, however well she might superintend the progress of the work. This plan, if a right one at all, should surely be modified; and the addition of a sighted person to correct work, and to teach the children nice habits, even apart from the fact that it is by the expression of the face that one judges whether the child is interested or not, would be an improvement on the present system, with which we have reason to believe the Board will not long remain satisfied.[1]

Schools for special instruction have now been opened in every division except the City; and in several districts experiments in boarding out groups of children near a centre are being carried out with great success. The work has progressed well, and has been favourably reported upon by H.M. Inspectors in each division. During last year, besides the ordinary rudiments of instruction, 332 boys and 349 girls, a total of 681, from the special centres have been taught in the schools of cookery; and 187 boys and 321 girls (total 508) have received instruction in laundry work. Forty-two girls have passed into the housewifery classes, and 319 boys into the manual training classes. It is hoped that many of the boy-cooks will find employment in the kitchens of hotels, etc. Fifty-three boys and 30 girls (total 83) who left school during the year are now wage-earners, and some parents who are keeping their girls at home testify to the useful and ready help they receive from them.

The Board makes arrangements for the safe transit to and from school of those children who are unable to travel by themselves, and in the case of the cripple schools, a nurse with an ambulance is in attendance, so that parents are left with no excuse for keeping their afflicted children at home, and they are waking up to the fact that it is "worth while" to allow them to be educated as far as their capacities allow.

[1] Four sighted teachers have, we understand, just been appointed, and will begin their work in the New Year.

